# Effects of the Non-Alcoholic Fraction of Beer on Abdominal Fat, Osteoporosis, and Body Hydration in Women

**DOI:** 10.3390/molecules25173910

**Published:** 2020-08-27

**Authors:** Marta Trius-Soler, Arnau Vilas-Franquesa, Anna Tresserra-Rimbau, Gemma Sasot, Carolina E. Storniolo, Ramon Estruch, Rosa M. Lamuela-Raventós

**Affiliations:** 1 Department of Nutrition, Food Sciences and Gastronomy, XaRTA, School of Pharmacy and Food Sciences, University of Barcelona, 08028 Barcelona, Spain; mtrius@ub.edu (M.T.-S.); annatresserra@ub.edu (A.T.-R.); gemmamsf@gmail.com (G.S.); carolina.e.storniolo@gmail.com (C.E.S.); 2 INSA-UB, Nutrition and Food Safety Research Institute, University of Barcelona, 08921 Santa Coloma de Gramanet, Spain; 3 CIBER Fisiopatología de la Obesidad y Nutrición (CIBEROBN), Instituto de Salud Carlos III, 28029 Madrid, Spain; restruch@clinic.cat; 4 Centre d’Innovació, Recerca i Transferència en Tecnologia dels Aliments (CIRTTA), XaRTA, TECNIO, MALTA-Consolider, Departament de Ciència Animal i dels Aliments, Facultat de Veterinària, Universitat Autònoma de Barcelona (Cerdanyola del Vallès), 08193 Bellaterra, Spain; arnauvilas.nl@gmail.com; 5 Department of Internal Medicine, Hospital Clínic, Institut d’Investigacions Biomèdiques August Pi i Sunyer (IDIBAPS), University of Barcelona, 08036 Barcelona, Spain

**Keywords:** hops, malt, health, menopause, polyphenol, phytoestrogen, prenylnarigenin, humulones, ethanol, bioactives

## Abstract

Several studies have shown that binge drinking of alcoholic beverages leads to non-desirable outcomes, which have become a serious threat to public health. However, the bioactive compounds in some alcohol-containing beverages might mitigate the negative effects of alcohol. In beer, the variety and concentration of bioactive compounds in the non-alcoholic fraction suggests that its consumption at moderate levels may not only be harmless but could also positively contribute to an improvement of certain physiological states and be also useful in the prevention of different chronic diseases. The present review focuses on the effects of non-alcoholic components of beer on abdominal fat, osteoporosis, and body hydration in women, conditions selected for their relevance to health and aging. Although beer drinking is commonly believed to cause abdominal fat deposition, the available literature indicates this outcome is inconsistent in women. Additionally, the non-alcoholic beer fraction might improve bone health in postmenopausal women, and the effects of beer on body hydration, although still unconfirmed seem promising. Most of the health benefits of beer are due to its bioactive compounds, mainly polyphenols, which are the most studied. As alcohol-free beer also contains these compounds, it may well offer a healthy alternative to beer consumers.

## 1. Introduction

Beer, an alcoholic drink composed of four main ingredients (water, malt, hops, and yeast) [1], is one of the most consumed beverages in the world [2]. From a nutritional point of view, its main components are water (around 90%), followed by carbohydrates, ethanol, minerals, vitamins, and bioactive compounds such as polyphenols and organic acids (iso-α-humulones). Beer composition, as well as its flavor, taste, and texture, differs considerably according to the ingredients and processing techniques [3]. Besides their health benefits, the bioactive compounds are also linked to the sensory characteristics of beer [4]. 

In view of the worldwide growth in beer consumption, studies investigating possible links between beer and different health outcomes are of utmost importance. Among others (i.e., liver disease), recently, one of the most important consequences of a high beer consumption is a greater risk of developing different site-specific cancers (e.g., colorectal [5], lung [6,7], prostate [8], and oral cavity, esophagus, and larynx cancer [9]). It is also known that high alcohol intake help to develop a dilated cardiomyopathy and also may trigger certain cardiovascular events [10,11]. Nevertheless, a moderate consumption of beer may also help to prevent these type of events [12,13]. 

Clinical evidence about beer consumption effects needs to be more specific on sex-related differences and health outcomes. Postmenopausal women due to the estrogen depletion suffer body changes [14] and there is an accumulation of abdominal fat [15], an increasing risk of osteoporosis [16] and a loss of body hydration [14] among other health issues. Interestingly, some studies have pointed out that bioactive compounds of beer may help to mitigate some of these adverse effects. 

In a unit of beer the main bioactive compounds with health benefits described in several studies [9,17,18] are depicted in Table 1. Particular attention has been given to the polyphenols found in malt (75%) and hops (25%), due to their antioxidant and anti-inflammatory properties [19,20]. Polyphenols are also critical to the flavor, astringency, bitterness, haze, and body of beer [21,22], and their concentration varies according to the ingredients and processing [23,24]. Regular beer, both ale and lager beers, is richer in polyphenol content compared to alcohol-free beers [25].

Among polyphenols, a particular group has attracted special interest for their estrogen-like properties [29]. Hops (*Humulus lupulus* L.) are a source of prenylflavonoids, a class of phytoestrogens, predominantly xanthohumol (XN), that during the brewing process isomerizes into isoxanthohumol (IX), 6-prenylnaringenine (6-PN), and 8-prenylnaringenine (8-PN) [30]. These compounds can mimic and modulate the action of estrogenic hormones by epigenetic mechanisms, via binding with cell surface receptors or by interacting with estrogen receptors (ERs). In particular, 8-PN has been described as the most estrogenic phytoestrogen, surpassing those typically found in soya products [31]. 

The aim of the present review is to summarize the available literature on the health outcomes of beer consumption in women, focusing on three specific health-related conditions: increased abdominal fat, osteoporosis, and overall body hydration. In particular, findings related to the beer bioactive compounds are discussed. 

## 2. Beer Consumption Related to Health and Disease in Women

### 2.1. Beer, Abdominal Fat, and Weight Gain 

A widely held belief is that beer consumption directly contributes to an increase in abdominal fat and ultimately leads to overweight and obesity. This assumption might be due to the nutritional value of beer, since it contains not only alcohol but also more carbohydrates than other alcoholic drinks [32]. In this section, we assess whether or not beer consumption can increase abdominal fat and site-specific adiposity in women, central obesity being the most relevant sign of metabolic syndrome (MetS) [33]. 

The type of alcoholic drink, as well as dose, frequency and time of consumption play a role in how alcohol drinking may change fat distribution [34,35]. Additional factors such as genetics, gender, and age may also be important determinants of central body fat [34]. Thus, for instance, drinking alcoholic beverages during meals was significantly more prevalent in females than in males in one study population [35]. In addition, it has been suggested that enlarged waist circumference (WC), known as “beer belly”, commonly observed in regular beer consumers might be more due to unhealthy lifestyle factors and drinking patterns (e.g., physical inactivity and smoking) rather than to beer consumption alone [36]. 

Women seem to be more prone to fat deposition than men upon the consumption of high doses of alcohol [37]. In general, postmenopausal women have a higher total body fat mass and more abdominal fat than premenopausal women. More specifically, despite exhibiting a similar mean body mass index (BMI), postmenopausal women have a larger WC [15]. While both genders experience somatic changes with aging, in women they particularly affect the WC and waist-to-hip ratio (WHR) [33,38]. Interestingly, both visceral and subcutaneous adipocytes express estrogen and androgen receptors such as ER-α, a regulator of adipocyte activity and fat distribution responsible for these gender differences and hyperandrogenism in postmenopausal women [15,39]. As increased visceral abdominal fat deposition causes metabolic changes in fatty acid metabolism, it would be useful to know which foods and ingredients may be more effective for counteracting this fat accumulation in postmenopausal women [15]. 

Several studies have investigated the effects of gender in the relationship between beer consumption and abdominal adiposity [32,40]. A systematic review of observational studies published before November 2010 indicates that there is an inverse or no association between general obesity and moderate beer consumption in women, while findings referring to abdominal obesity seem to be inconsistent [40]. The authors pointed out that these conflicting observational data may be explained by the small proportion of women beer drinkers and their relatively low beer intake in the studies analyzed [40]. 

Alcohol or beer consumption and abdominal fat or weight gain have been described as having a U-shaped relationship, with the lowest BMI values observed in women who consumed an average of 6–24 g/day of alcohol [41]. In another study, women with a low beer consumption (maximum 1.32 L/week) also had the lowest WHR values, whereas non-consumers had the highest WC [33]. In the Third National Health and Nutrition Examination Survey (NHANES III), the lowest MetS and WC values were observed in the mild to moderate beer and wine drinkers [42]. Consequently, it can be stated that excessive beer intake may contribute to a higher WC and WHR, and even a higher overall BMI, yet the regular consumption of less than 0.5 L/day of beer (4% alcohol) seems unlikely to have this effect, according to the data available in cross-sectional and prospective observational studies [40]. Women studies evaluating the relationship between beer consumption and abdominal fat increase has been summarized in Table 2 [33,35,36,37,41,43,44,45,46,47,48,49,50,51,52,53,54,55].

In a study focused on the effects of a moderate beer intake on the body composition of healthy adults undergoing a high-intensity interval training, the group consuming alcohol-free beer experienced a significant decrease in visceral adipose tissue and WC, and a clear decreasing trend in the WHR. The other groups (consuming beer or water supplemented with vodka ethanol) did not show any changes in these variables [56]. 

Now, we should look for the compounds of regular and non-alcoholic beer responsible of these effects. The main bitter compounds of beer are iso-α-acids or iso-α-humulones, derived from the isomerization of α-acids in hops during brewing [57,58]. A study of mice fed with a high-fat diet (HFD) supplemented with iso-α-acids reported significantly reduced body weight, epididymal fat weight, and plasma triglyceride levels after the intervention, whereas in the control group the values increased [59]. As in other studies, it was concluded that iso-humulones might have a protective effect on internal organs damaged by obesity, making this a promising line of future research [59,60]. Iso-α-acids bind and activate both peroxisome proliferator-activated receptors α (PPARα) and γ (PPARγ), which exhibit anti-obesity and anti-inflammatory activities in vivo [59,60,61]. Regular beers contain 20–40 mg/L of iso-α-acids [27,62,63], and some bitter beers up to 50–80 mg/L [62]. 

A clinical trial with prediabetes subjects found that 32–48 mg/day of iso-humulones lowered the fasting blood glucose and hemoglobin A1c after 8 weeks, while the total fat and BMI in participants receiving 48 mg/day decreased at 12 weeks [62]. However, some effective concentrations of iso-humulones reported in the literature, such as 500 mg/kg body weight in mice, would be impossible to ingest through moderate or even high beer consumption [60]. Additionally, it would be difficult to formulate a food other than beer with 10–100 mg/L of iso-humulones and an effective dose of iso-α-acids because of their strong bitterness [57].

Matured hop bitter acids (MHBA) are components derived from α-acid oxidation and bear a β-tricarbonyl moiety in their structure such as α-, β-, and iso-α-acids. The bitterness of α-acid oxidation products is described as being more acceptable for the consumer compared to iso-α-acids, and some studies of the bioactive properties of MHBA have been carried out [57]. Weight gain in six-week-old male C57BL/6J mice, a model of MetS, was significantly suppressed when their high fat diet was supplemented with MHBA [64]. Additionally, MHBA administration induced cholecystokinin secretion and signal transduction in the rat gastrointestinal tract, resulting in an increase in the brown adipose tissue temperature. Moreover, MHBA may target TAS2 receptors (TAS2Rs) because they share a similar structure with iso-α-acid [57]. Although 25 TAS2 bitter taste receptors have been determined in humans, only TAS2R1, TAS2R14, and TAS2R40 have been reported to mediate psychophysical responses to bitter hop-derived compounds [65]. Specifically, TAS2R1 and TAS2R40 are expressed in enteroendocrine cells, responsible for incretin hormone secretion [66,67,68]. There is also interesting evidence that the consumption of mature hop extract significantly reduces abdominal visceral fat of healthy overweight subjects [58]. 

On the other hand, it has been found that a XN-rich hop extract (17.8% XN and 12.4% IX) prevents fat gain due to overnutrition by modulating preadipocyte differentiation in a 3T3-L1 mouse fibroblast cell line [69]. Furthermore, oral administration of 30 and 60 mg/kg/day of XN during 12-weeks in a C57BL/6J mice model improved markers of inflammation and MetS and decreased BMI in a dose-dependent manner. Nevertheless, the authors concluded that because XN concentrations found in beer are only about 0.2 mg/L, XN taken in the form of beer would be unlikely to have a protective effect against MetS [70]. Two other studies performed in the same C57BL/6J mice model demonstrated that XN derivatives [71] and IX [72] significantly changed the gut microbiota profile, constituting a potential mechanism against obesity and MetS [71,72]. 

### 2.2. Beer and Osteoporosis

Known as one of the most important health-related conditions of aging, osteoporosis is attributed to a decrease of bone mineral density (BMD), which ultimately leads to increased bone fragility [73]. Although common, the condition is underdiagnosed and undertreated, and clinical trials and public health strategies are needed to improve screening and management [74]. Nutrition, exercise and lifestyle are recognized as important aspects in osteoporosis prognosis [75], so modifiable environmental factors such as diet should be considered in its management [76].

Postmenopausal status has been described as a risk factor of BMD loss [16]. As a long-term consequence of the lack of estrogenic stimulation, menopausal bone loss has been linked to an accelerated bone turnover combined with an imbalance that favors bone resorption rather than formation [29,77]. The risk of osteoporosis is six times higher in postmenopausal versus premenopausal women [74]. One of the main mechanisms underlying the protective effect of estrogen against osteoporosis could be an enhanced expression of the vitamin D receptor in the duodenal mucosa and responsiveness to endogenous 1,25-dihydroxycolecalciferol [78].

Certain dietary factors, such as moderate alcohol consumption, have been positively associated with BMD values in postmenopausal women and in the general population [16,79,80]. A study found that women who consumed more than 1 drink of alcohol/day (i.e., 270 mL of beer, 100 mL of wine, or 27 mL of liquor) had a significantly higher femoral neck and lumbar spine BMD than non-alcohol consumers, in a lifestyle adjusted model [81]. Among alcoholic drink subtypes, only beer and low-alcohol beer (but not wine or liquors) seemed to have a significantly positive effect on lumbar spine BMD in older women [81,82]. Similarly, in a cohort of elderly men and women, the lowest hazard ratios for hip fracture tended to be among beer consumers [83]. Also, quantitative bone ultrasound values were higher in women who consumed beer compared to the non-beer or wine drinkers, independently of their gonadal status. This result could be explained by the phytoestrogen content and low grade of alcohol in beer [84]. In contrast, other studies have found positive associations between wine or wine preference and spine BMD in a postmenopausal population group, but not for beer or spirits [76,85]. Women studies evaluating the relationship between beer consumption and osteoporosis has been summarized in Table 3 [76,81,82,84].

In 2008, a systematic review and meta-analysis concluded that subjects consuming 0.5–1 drink/day, equivalent to 7–14 g alcohol/day, had a lower hip fracture risk than abstainers, whereas those consuming more than 2 drinks/day had a greater risk [86]. Thus, abstainers and heavy drinkers have a higher risk of hip fractures than light-moderate drinkers, with a U-shaped relationship between the variables [83,86]. Supporting these results, abnormal bone histology and decreasing bone formation and mineralization have been described in alcoholics [87]. The tendency of a higher association between BMD and beer or wine consumption compared to liquor suggests that other compounds besides ethanol may contribute to bone health [4]. 

Most of the positive effects of beer on osteoporosis in postmenopausal women have been attributed to the non-alcoholic fraction, specifically to polyphenols, silicon and α-acids. Among phenolic compounds, flavonoids have been inversely linked to bone resorption biomarkers in Scottish women aged 45–54 years. The flavonoids most consumed by the participants were catechins, demonstrating the significant contribution of these compounds to improving BMD [88,89]. The bioactive compounds in hops have been proposed as an alternative to conventional hormone replacement therapy. In particular, the phenolic phytoestrogens from hop extract seem to exhibit estrogen-like effects on bone metabolism [90]. A recent study in animals found that hop extract containing phytoestrogens and iso-α-acids attenuated bone loss and reversed high bone turnover in ovariectomy mice [91]. Furthermore, in vitro experiments demonstrate that hop phytoestrogens (XN, IX, 6-PN, and 8-PN) regulate both osteoblast and osteoclast activities, while α-acids exert a strong bone resorption inhibitory activity, however, the recommended dosage is still unclear [90,91,92]. 

The phytoestrogen XN inhibits the receptor activator for the nuclear factor κ B ligand (RANKL) signaling pathway, which has been identified as critical to osteoclast formation and bone resorption [93,94]. XN has also been reported to promote osteoblast differentiation, up-regulate alkaline phosphatase activity, and increase the expression of osteogenic marker genes in osteoblastic cell lines [95]. Interestingly, Prouillet et al. (2004) had previously suggested that one of the consequences of increased alkaline phosphatase activity could be an activation of the ER [94], and another study described an inhibitory resorption effect of XN in a dose-dependent manner [92]. Regarding 8-PN, a recent review of its therapeutic perspectives discusses plausible mechanisms for the anti-osteoporotic properties of this intestinal metabolite. 8-PN has preferential binding to ER-α, which is the prevailing ER in bone tissue, and its prenyl group seems to be essential for the anti-osteoporotic mechanism [29]. In summary, the beneficial effects of 8-PN, promoting bone formation and inhibiting bone resorption, are mediated by ER-α instead of ER-β, and it is more potent than the isoflavones genistein and daidzein [96].

Silicon from malt has been reported to facilitate bone mineralization and regeneration [75,97], which are essential for bone formation [97]. Some alcoholic beverages such as beer or wine contain significant amounts of silicon [98], although due to the processing of barley and hops, beer is a better source than wine or other alcoholic beverages, with an average content of 19.2 mg/L and non-significant differences among different types of beer [28,75]. Moreover, silicon in beer has a high bioavailability [98,99]. Tucker et al. (2009) showed that adjustment for silicon intake mitigates the positive effect of beer consumption on BMD in older men and women [4]. 

To sum up, bone remodeling is a slow process and aging affects bone turnover [100]. The phenolic fraction of beer, including phytoestrogens and iso-α-acids from hops, and the silicon from malt seem to play a role in osteoporosis prevention. However, long-term clinical trials are needed to better predict the impact of beer consumption on bone mass, a major concern for postmenopausal women suffering from bone loss. 

### 2.3. Beer and Body Hydration

Hydration has a crucial impact on a variety of factors related to the correct functioning of the body and specific recommendations are needed for each population group. Female sex hormones affect the body water balance, although it is still unclear how the regulation of hydration in women may enhance wellness, safety, and mental and physical performance [101]. Estrogen and progesterone levels have been correlated with body fluid regulation and thermoregulation changes [101]. As more water is retained in the body when estrogen levels are high [102], hormonal depletion in menopause results in a loss of hydration, which should be carefully monitored. Current literature reports that estrogen therapy increases osmotic sensitivity and water retention, helping menopausal women to control diuresis and prevent dehydration [14]. The effect of estrogen on fluid regulation in older women seems to be related to sodium retention [102,103]. Not only the menopause but aging itself affects the fluid balance [14]. 

An estimated intake of 2.5 L of water/day is considered necessary under normal conditions or 3.5 L of water/day in hot weather or when exercising [104]. Perspiration while exercising may cause an important depletion of water and electrolytes [105], as well as part of the body’s stored glycogen. Most recommendations for sustaining the nutritional state and optimizing water absorption during exercise include the intake of beverages containing carbohydrates and electrolytes, in particular glucose–fructose and sodium [106]. Besides the main components of water and carbohydrates, beer also contains electrolytes, which may play a role in maintaining water and electrolyte balance, although the ethanol content may counteract these positive effects.

The effect of beer consumption on the overall hydration status has been studied among men. Unfortunately, no studies on this issue have been performed in women. Hobson and Maughan (2010) investigated the effect of low-alcohol doses on induced euhydration or hypohydration [107], administering alcohol-free or alcoholic beer in each case to create four experimental conditions. In the euhydrated group, those consuming alcoholic beer produced more total urine in the 4 h after intake and for 3 h also exhibited considerably higher serum osmolality, a parameter associated with fluid balance, although the difference had disappeared at 4 h, the end of the monitoring period. The authors also mentioned that sodium excretion was notably lower in the alcohol consumers [108]. In an elderly population with more hydration problems, Polhuis et al. (2017) observed a temporary diuretic effect only after moderate consumption of stronger alcoholic beverages (wine, spirits), but not beer. This demonstrates that: (i) moderate consumption of beer and other weak alcoholic beverages may be safe in terms of hydration for the elderly and (ii) the diuretic effect was plainly triggered by the amount of alcohol in the beverage [108].

Several studies have investigated the effect of beer or its components in those practicing sports, monitoring hydration status, muscle performance, environmental conditions, and duration of exercise in male athletes [105,109,110]. The most controversial component of beer is ethanol. An early study from 1997 reported that the retention volume of the total fluid ingested was about 20% lower in those who consumed an alcohol-free beer supplemented with 4% alcohol compared to those who drank non-supplemented alcohol-free beer, following intermittent cycle ergometer exercises in the heat that induced dehydration of up to 2% of body mass [111]. Alcohol itself undoubtedly has a negative effect on exercise performance, although its extent may also depend on other factors, such as the mode and duration of exercise [109]. In extreme conditions, when the body requires greater hydration, any diuretic or anti-hydration effect of the ethanol in beer is more easily noted. Jiménez-Pavón et al. (2015) observed that consumption of 660 mL of regular beer (4% alcohol) after 1 h of running in hot conditions had no deleterious effect on any hydration marker [106]. Two other studies evaluated the effect of water, beer or alcohol-free beer on fluid and electrolyte homeostasis in male athletes or physically active men [112,113]. Castro-Sepulveda et al. (2016) reported that an intake of 700 mL of alcoholic beer before aerobic exercising increased plasma K^+^ and decreased plasma Na^+^ during the exercise activity, with a negative impact on athletic performance. Notably, this effect was not observed when alcohol-free beer was administered, to the extent that the decrease in plasma Na^+^ during exercise was lower than after the ingestion of water. Accordingly, alcohol-free beer might be an effective sports drink for maintaining electrolyte homeostasis in males when taken before exercise [113]. In contrast, another study found that rehydration of young, healthy, and physically active males with non-alcoholic beer was not advantageous with regard to water [112]. A more recent study evaluated the effects of ingesting isotonic drinks or beer with different alcohol concentrations after mild dehydration or exercise among males. The net fluid balance was measured after a 5-hour observation period and the lowest rate of fluid retention (21%) was obtained for beer with 5% alcohol, whereas the highest (42%) was recorded for an isotonic sports drink [114]. Interestingly, the effects of modifying the sodium and alcohol content of beer have also been studied [115,116]. Participants consumed low-alcohol beer (2% alcohol + 25 or 50 mM/L of sodium) or normal beer (3.5% alcohol + 25 mM/L of sodium) and after exercise, the greatest fluid retention was observed in consumers of beer with the highest electrolyte content and the lowest concentration of alcohol (2% alcohol + 50 mM/L of sodium) [116]. 

While non-alcoholic beer has promising effects in terms of fluid homeostasis in the context of aerobic exercise, a low dose of alcohol (0.5 g/kg of body weight) consumed before muscle damage-inducing anaerobic exercise had no impact on the posterior muscle performance or related water loss in ten healthy young males [110]. 

Notably, all the aforementioned studies were performed in men. More research is needed to understand the effects of different types of drinks on the hydration state of female athletes, in order to improve performance and provide personalized supplementation recommendations [101]. 

## 3. Implications and Future Research

Most of the health benefits of beer are thought to be originated by its non-alcoholic components, mainly polyphenols. Although found in small quantities in the final product, the flavonoid XN (whose only source is hops) is of particular interest. Intestinal metabolites of related flavonoids, notably 8-PN, could also have an important role in human health. Other components, such as silicon or bitter acids, may help to explain other health effects of beer consumption, such as improvement in bone density. Nevertheless, the beneficial properties of beer components outlined in this review have not been extensively studied because of the adverse effects of ethanol. Human interventional trials are required to elucidate the real association between beer intake and health benefits in women, but the consumption of ethanol is an important obstacle for their development. We, therefore, suggest a directional change towards the non-alcoholic fraction of beer and its effect on the female population as an interesting target for future studies. With some authors already using this strategy, a greater focus on alcohol-free beer will lead to the emergence of more human trials and new evidence in this field. Finally, new long-term randomized trials on the effects of moderate alcoholic and non-alcoholic beer consumption (and other alcoholic beverages) on health and diseases, including cardiovascular disease, obesity, diabetes, cancer, cognitive decline, osteoporosis, and others in women (and also in women) are needed to better define the protective role (or not) of beer consumption, independent of other lifestyle factors, on the aforementioned conditions.

## 4. Conclusions

Although the results of studies on abdominal fat deposition in female beer consumers are inconsistent, moderate consumption appears not to have a significant effect on adiposity. Moderate beer intake has also been associated with improved bone health in elderly women in observational studies. Moreover, the non-alcoholic fraction of beer is of potential interest as a counteracting agent for bone mass loss after menopause. 

In the elderly, beer intake does not seem to pose a risk for hydration. When ingested before exercise, beer with lower alcohol content has a better rehydration effect, and the consumption of alcohol-free beer may even have a positive impact on electrolyte homeostasis. However, the effects of beer on hydration in women still need to be investigated.

## Figures and Tables

**Table 1:** Mean content of selected bioactive compounds in a standard drink of regular beer.

Bioactive Compound	Avarege Level (mg/330 mL)
**Phytoestrogens** Xanthohumol 6-Prenylnaringenin 8-Prenylnaringenin Isoxanthohumol	4.653 × 10^−3^ 8.547 × 10^−3^ 3.432 × 10^−3^ 0.132
**Bitter acids** α+β acids Iso-α-humulones **Minerals** Silicon Sodium Potassium	0.891 ^a^ 9.207 ^a^ 6.336 14.883 116.589

^a^ mean value from three beer samples. Content of phytoestrogens from Rothwell et al. (2013) [26], bitter acids from Česlová et al. (2009) [27], silicon from Jugdaohsingh (2007) [28] and sodium and potassium derived from *the Food composition data of 16 European countries* via www.EuroFIR.org.

**Table 2:** Women studies evaluating the relationship between beer consumption and abdominal fat increase.

Authors Year [Ref]	Type of Study	Study Population	Key Finding
Lapidus et al., 1989 [43]	Cross-sectional	1462 women 38–60 years-old	No correlation was found between WHR and beer consumption.
Slattery et al., 1992 [44]	Cross-sectional	1447 black women 1284 white women 18–30 years-old	Higher beer consumption was associated with a higher WHR among white and black women.
Kahn et al., 1997 [45]	Prospective observational	44080 women 40–54 years-old	OR of abdominal weight gain was positively associated in women drinking >0 to <5 days per week and no associated in women drinking <5 days per week versus non-drinkers
Dallongeville et al., 1998 [37]	Cross-sectional	11730 women 35–64 years-old	Beer & cider consumption was associated with a higher WHR.
Rosmond & Bjorntorp 1999 [46]	Cross-sectional	1137 women 40 years-old	Beer consumption was negatively correlated to WHR.
Machado & Sichieri 2002	Cross-sectional	1396 women 20–60 years-old	No trend association for OR for WHR >0.80 across beer consumption categories was found.
Vadstrup et al., 2003 [48]	Prospective observational	3970 women 20–83 years-old	Positive trend association was found for WC at follow-up across beer intake categories.
Bobak et al., 2003 [49]	Cross-sectional	1098 women 25–64 years-old	Beer intake was not associated with an increase in WHR.
Dorn et al., 2003 [35]	Cross-sectional	1322 women 53.3 ± 9.4 years-old	No trend association was found between sagittal abdominal diameter and beer consumption.
Halkjaer et al., 2004 [50]	Prospective observational	1131 women 30–60 years-old	Women consuming >4 drinks of beer per week have higher WC, while no significance increase in WC was found in the group drinking 1–3 drinks of beer per week compared to non-drinkers.
Deschamps et al., 2004 [52]	Cross-sectional	284 women 42.4 ± 4.6 years-old	Women drinking >1 glass of beer per day have a higher WRC than abstainers and those who drink <1 glass of beer per day. No trend association was found for WC.
Lukasiewicz et al., 2005 [53]	Cross-sectional	1268 women 47.7 ± 6.6 years-old	No trend association was found between beer consumption and WHC.
Halkjaer et al., 2006	Prospective observational	22570 women 55 (50–64) years-old	No trend association was found between ΔWC and beer consumption.
Krachler et al., 2006 [54]	Cross-sectional	3087 women 25–64 years-old	Increased beer consumption was not significantly associated to WC.
Tolstrup et al., 2008 [55]	Prospective observational	1610 women 50–65 years-old	Negative association was found for OR of WC across beer intake frequency categories among women who preferred beer.
Schütze et al. [36] 2009	Cross-sectional	2749 women 35–65 years-old	Positive trend association for ΔWC and ΔWHR was found across beer consumption categories.
Schütze et al., 2009 [36]	Prospective observational	12749 women 35–65 years-old	No trend association for WC was found across beer consumption categories.
Bergmann et al., 2011 [41]	Cross-sectional	158796 women 52.9 ± 9 years-old	Positive association was found for OR of WC and WHR for women drinking <6 versus ≤ 6 g per day of alcohol from beer.
Zugravu et al., 2019 [33]	Cross-sectional	784 women >18 years-old	No linear trend association was found between beer consumption and WC or WHR.

WC: waist circumference; WHR: waist-hip ratio.

**Table 3:** Women studies evaluating the relationship between beer consumption and osteoporosis.

Authors Year [Ref]	Type of Study	Study Population	Key Finding
Pedrera-Zamorano et al., 2009 [86]	Cross-sectional	1697 women (710 premenopausal; 176 perimenopausal and 811 postmenopausal) 48.8 ± 12.59 years-old	Light or moderate consumption of beer was associated to higher bone mass in women independently on their gonadal status.
Fairweather-Tait et al., 2011 [76]	Cross-sectional	2464 postmenopausal women twins 56.3 ± 11.9 years-old	Beer consumption was not associated with higher BMD.
Yin et al., 2011 [82]	Cross-sectional	428 women 62.6 ± 7.2 years-old	Low alcohol beer consumption frequency was positively associated with BMD at lumbar spine.
Yin et al., 2011 [82]	Prospective observational	428 women 62.6 ± 7.2 years-old	No association between beer consumption frequency and BMD at hip was found.
McLenon et al., 2012 [81]	Prospective observational	3173 women 50–62 years-old	Moderate beer consumption had a positive significant effect on lumbar spine BMD after adjustment for lifestyle.
Kubo et al., 2013 [85]	Prospective observational	115,655 postmenopausal women 50–79 years-old	No association was observed between ≥ 1 servings of beer per week and risk of hip fracture.

BMD: bone mineral density.

## References

[1] Buiatti S. (2009). Beer Composition: An Overview. Beer in Health and Disease Prevention.

[2] Colen L., Swinnen J. (2016). Economic growth, globalisation and beer consumption. J. Agric. Econ..

[3] Stewart G.G., Priest F.G. (2006). Handbook of Brewing.

[4] Tucker K.L., Jugdaohsingh R., Powell J.J., Qiao N., Hannan M.T., Sripanyakorn S., Cupples L.A., Kiel D.P. (2009). Effects of beer, wine, and liquor intakes on bone mineral density in older men and women. Am. J. Clin. Nutr..

[5] Zhang C., Zhong M. (2015). Consumption of beer and colorectal cancer incidence: A meta-analysis of observational studies. Cancer Causes Control.

[6] Benedetti A., Parent M.E., Siemiatycki J. (2006). Consumption of alcoholic beverages and risk of lung cancer: Results from two case-control studies in Montreal, Canada. Cancer Causes Control.

[7] Chao C. (2007). Associations between beer, wine, and liquor consumption and lung cancer risk: A meta-analysis. Cancer Epidemiol. Biomark. Prev..

[8] Demoury C., Karakiewicz P., Parent M.-E. (2016). Association between lifetime alcohol consumption and prostate cancer risk: A case-control study in Montreal, Canada. Cancer Epidemiol..

[9] de Gaetano G., Costanzo S., Di Castelnuovo A., Badimon L., Bejko D., Alkerwi A., Chiva-Blanch G., Estruch R., La Vecchia C., Panico S. (2016). Effects of moderate beer consumption on health and disease: A consensus document. Nutr. Metab. Cardiovasc. Dis..

[10] Reynolds K., Lewis L.B., Nolen J.D.L., Kinney G.L., Sathya B., He J. (2003). Alcohol consumption and risk of stroke: A meta-analysis. J. Am. Med. Assoc..

[11] Mukamal K.J. (2008). Alcohol, beer, and ischemic stroke. Beer in Health and Disease Prevention.

[12] Costanzo S., Di Castelnuovo A., Donati M.B., Iacoviello L., de Gaetano G. (2011). Wine, beer or spirit drinking in relation to fatal and non-fatal cardiovascular events: A meta-analysis. Eur. J. Epidemiol..

[13] Di Castelnuovo A., Rotondo S., Iacoviello L., Donati M.B., De Gaetano G. (2002). Meta-analysis of wine and beer consumption in relation to vascular risk. Circulation.

[14] Stachenfeld N.S. (2014). Hormonal changes during menopause and the impact on fluid regulation. Reprod. Sci..

[15] Ko S.H., Kim H.S. (2020). Menopause-associated lipid metabolic disorders and foods beneficial for postmenopausal women. Nutrients.

[16] Bainbridge K., Sowers M., Lin X., Harlow S. (2004). Risk factors for low bone mineral density and the 6-year rate of bone loss among premenopausal and perimenopausal women. Osteoporos. Int..

[17] Sripanyakorn S., Jugdaohsingh R., Mander A., Davidson S.L., Thompson R.P.H., Powell J.J. (2009). Moderate ingestion of alcohol is associated with acute ethanol-induced suppression of circulating CTX in a PTH-independent fashion. J. Bone Miner. Res..

[18] Arranz S., Chiva-Blanch G., Valderas-Martínez P., Medina-Remón A., Lamuela-Raventós R.M., Estruch R. (2012). Wine, beer, alcohol and polyphenols on cardiovascular disease and cancer. Nutrients.

[19] Proestos C., Komaitis M. (2008). Antioxidant Capacity of Hops.

[20] Feick P., Gerloff A., Singer M.V. (2009). The effect of beer and its non-alcoholic constituents on the exocrine and endocrine pancreas as well as on gastrointestinal hormones. Beer in Health and Disease Prevention.

[21] Callemien D., Collin S. (2010). Structure, organoleptic properties, quantification methods, and stability of phenolic compounds in beer—A review. Food Rev. Int..

[22] Intelmann D., Haseleu G., Dunkel A., Lagemann A., Stephan A., Hofmann T. (2011). Comprehensive sensomics analysis of hop-derived bitter compounds during storage of beer. J. Agric. Food Chem..

[23] Rivero D., Pérez-Magariño S., González-Sanjosé M.L., Valls-Belles V., Codoñer P., Muñiz P. (2005). Inhibition of induced DNA oxidative damage by beers: Correlation with the content of polyphenols and melanoidins. J. Agric. Food Chem..

[24] Venturelli S., Burkard M., Biendl M., Lauer U.M., Frank J., Busch C. (2016). Prenylated chalcones and flavonoids for the prevention and treatment of cancer. Nutrition.

[25] Boronat A., Soldevila-Domenech N., Rodríguez-Morató J., Martínez-Huélamo M., Lamuela-Raventós R.M., de la Torre R. (2020). Beer phenolic composition of simple phenols, prenylated flavonoids and alkylresorcinols. Molecules.

[26] Rothwell J.A., Perez-Jimenez J., Neveu V., Medina-Remon A., M’Hiri N., Garcia-Lobato P., Manach C., Knox C., Eisner R., Wishart D.S. (2013). Phenol-Explorer 3.0: A major update of the Phenol-Explorer database to incorporate data on the effects of food processing on polyphenol content. Database.

[27] Česlová L., Holčapek M., Fidler M., Drštičková J., Lísa M. (2009). Characterization of prenylflavonoids and hop bitter acids in various classes of Czech beers and hop extracts using high-performance liquid chromatography-mass spectrometry. J. Chromatogr. A.

[28] Jugdaohsingh R. (2007). Silicon and bone health. J. Nutr. Health Aging.

[29] Štulíková K., Karabín M., Nešpor J., Dostálek P. (2018). Therapeutic perspectives of 8-prenylnaringenin, a potent phytoestrogen from hops. Molecules.

[30] Quifer-Rada P., Vallverdú-Queralt A., Martínez-Huélamo M., Chiva-Blanch G., Jáuregui O., Estruch R., Lamuela-Raventós R. (2015). A comprehensive characterisation of beer polyphenols by high resolution mass spectrometry (LC–ESI-LTQ-Orbitrap-MS). Food Chem..

[31] Omoruyi I.M., Pohjanvirta R. (2018). Estrogenic activities of food supplements and beers as assessed by a yeast bioreporter assay. J. Diet. Suppl..

[32] Wannamethee S.G. (2009). Beer and Adiposity.

[33] Zugravu C.-A., Pătrașcu D., Otelea M. (2019). Central obesity and beer consumption. Ann. Univ. Dunarea Jos Galati Fascicle VI Food Technol..

[34] Ferreira M.G., Valente J.G., Gonçalves-Silva R.M.V., Sichieri R. (2008). Alcohol consumption and abdominal fat in blood donors. Rev. Saude Publica.

[35] Dorn J.M., Hovey K., Muti P., Freudenheim J.L., Russell M., Nochajski T.H., Trevisan M. (2003). Alcohol drinking patterns differentially affect central adiposity as measured by abdominal height in women and men. J. Nutr..

[36] Schütze M., Schulz M., Steffen A., Bergmann M.M., Kroke A., Lissner L., Boeing H. (2009). Beer consumption and the “beer belly”: Scientific basis or common belief?. Eur. J. Clin. Nutr..

[37] Dallongeville J., Marécaux N., Ducimetière P., Ferrières J., Arveiler D., Bingham A., Ruidavets J., Simon C., Amouyel P. (1998). Influence of alcohol consumption and various beverages on waist girth and waist-to-hip ratio in a sample of French men and women. Int. J. Obes..

[38] Wong M.C.S., Huang J., Wang J., Chan P.S.F., Lok V., Chen X., Leung C., Wang H.H.X., Lao X.Q., Zheng Z.-J. (2020). Global, regional and time-trend prevalence of central obesity: A systematic review and meta-analysis of 13.2 million subjects. Eur. J. Epidemiol..

[39] Marchand G.B., Carreau A.-M., Weisnagel S.J., Bergeron J., Labrie F., Lemieux S., Tchernof A. (2018). Increased body fat mass explains the positive association between circulating estradiol and insulin resistance in postmenopausal women. Am. J. Physiol. Metab..

[40] Bendsen N.T., Christensen R., Bartels E.M., Kok F.J., Sierksma A., Raben A., Astrup A. (2013). Is beer consumption related to measures of abdominal and general obesity? A systematic review and meta-analysis. Nutr. Rev..

[41] Bergmann M.M., Schütze M., Steffen A., Boeing H., Halkjaer J., Tjonneland A., Travier N., Agudo A., Slimani N., Rinaldi S. (2011). The association of lifetime alcohol use with measures of abdominal and general adiposity in a large-scale European cohort. Eur. J. Clin. Nutr..

[42] Freiberg M.S., Cabral H.J., Heeren T.C., Vasan R.S., Curtis Ellison R. (2004). Alcohol consumption and the prevalence of the metabolic syndrome in the U.S.: A cross-sectional analysis of data from the Third National Health and Nutrition Examination Survey. Diabetes Care.

[43] Lapidus L., Bengtsson C., Hällström T., Björntorp P. (1989). Obesity, adipose tissue distribution and health in women-Results from a population study in Gothenburg, Sweden. Appetite.

[44] Slattery M.L., McDonald A., Bild D.E., Caan B.J., Hilner J.E., Jacobs D.R., Liu K. (1992). Associations of body fat and its distribution with dietary intake, physical activity, alcohol, and smoking in blacks and whites. Am. J. Clin. Nutr..

[45] Kahn H.S., Tatham L.M., Heath C.W. (1997). Contrasting factors associated with abdominal and peripheral weight gain among adult women. Int. J. Obes..

[46] Rosmond R., Björntorp P. (1999). Psychosocial and socio-economic factors in women and their relationship to obesity and regional body fat distribution. Int. J. Obes..

[47] Machado P.A.N., Sichieri R. (2002). Relação cintura-quadril e fatores de dieta em adultos. Rev. Saude Publica.

[48] Vadstrup E., Petersen L., Sørensen T., Grønbaek M. (2003). Waist circumference in relation to history of amount and type of alcohol: Results from the Copenhagen City Heart Study. Int. J. Obes. Relat. Metab. Disord..

[49] Bobak M., Skodova Z., Marmot M. (2003). Beer and obesity: A cross-sectional study. Eur. J. Clin. Nutr..

[50] Halkjær J., Sørensen T.I., Tjønneland A., Togo P., Holst C., Heitmann B.L. (2004). Food and drinking patterns as predictors of 6-year BMI-adjusted changes in waist circumference. Br. J. Nutr..

[51] Halkjær J., Tjønneland A., Thomsen B.L., Overvad K., Sørensen T.I.A. (2006). Intake of macronutrients as predictors of 5-y changes in waist circumference. Am. J. Clin. Nutr..

[52] Deschamps V., Alamowitch C., Borys J. (2004). Boissons alcooliques, poids et paramètres d’adiposité chez 520 adultes issus de l’étude Fleurbaix Laventie Ville Santé. Cah. Nutr. Diététique.

[53] Lukasiewicz E., Mennen L.I., Bertrais S., Arnault N., Preziosi P., Galan P., Hercberg S. (2005). Alcohol intake in relation to body mass index and waist-to-hip ratio: The importance of type of alcoholic beverage. Public Health Nutr..

[54] Krachler B., Eliasson M., Stenlund H., Johansson I., Hallmans G., Lindahl B. (2006). Reported food intake and distribution of body fat: A repeated cross-sectional study. Nutr. J..

[55] Tolstrup J.S., Halkjær J., Heitmann B.L., Tjønneland A.M., Overvad K., Sørensen T.I.A., Grønbæk M.N. (2008). Alcohol drinking frequency in relation to subsequent changes in waist circumference. Am. J. Clin. Nutr..

[56] Molina-Hidalgo C., De-Lao A., Jurado-Fasoli L., Amaro-Gahete F.J., Castillo M.J. (2019). Beer or ethanol effects on the body composition response to high-intensity interval training. The BEER-HIIT study. Nutrients.

[57] Yamazaki T., Morimoto-Kobayashi Y., Koizumi K., Takahashi C., Nakajima S., Kitao S., Taniguchi Y., Katayama M., Ogawa Y. (2019). Secretion of a gastrointestinal hormone, cholecystokinin, by hop-derived bitter components activates sympathetic nerves in brown adipose tissue. J. Nutr. Biochem..

[58] Morimoto-Kobayashi Y., Ohara K., Ashigai H., Kanaya T., Koizumi K., Manabe F., Kaneko Y., Taniguchi Y., Katayama M., Kowatari Y. (2015). Matured hop extract reduces body fat in healthy overweight humans: A randomized, double-blind, placebo-controlled parallel group study. Nutr. J..

[59] Ayabe T., Ohya R., Kondo K., Ano Y. (2018). Iso-α-acids, bitter components of beer, prevent obesity-induced cognitive decline. Sci. Rep..

[60] Miura Y., Hosono M., Oyamada C., Odai H., Oikawa S., Kondo K. (2005). Dietary isohumulones, the bitter components of beer, raise plasma HDL-cholesterol levels and reduce liver cholesterol and triacylglycerol contents similar to PPARα activations in C57BL/6 mice. Br. J. Nutr..

[61] Dostálek P., Karabín M., Jelínek L. (2017). Hop phytochemicals and their potential role in metabolic syndrome prevention and therapy. Molecules.

[62] Obara K., Mizutani M., Hitomi Y., Yajima H., Kondo K. (2009). Isohumulones, the bitter component of beer, improve hyperglycemia and decrease body fat in Japanese subjects with prediabetes. Clin. Nutr..

[63] Vanhoenacker G., De Keukeleire D., Sandra P. (2004). Analysis of iso-α-acids and reduced iso-α-acids in beer by direct injection and liquid chromatography with ultraviolet absorbance detection or with mass spectrometry. J. Chromatogr. A.

[64] Morimoto-Kobayashi Y., Ohara K., Takahashi C., Kitao S., Wang G., Taniguchi Y., Katayama M., Nagai K. (2015). Matured hop bittering components induce thermogenesis in brown adipose tissue via sympathetic nerve activity. PLoS ONE.

[65] Intelmann D., Batram C., Kuhn C., Haseleu G., Meyerhof W., Hofmann T. (2009). Three TAS2R bitter taste receptors mediate the psychophysical responses to bitter compounds of hops (*Humulus lupulus* L.) and beer. Chemosens. Percept..

[66] Kok B.P., Galmozzi A., Littlejohn N.K., Albert V., Godio C., Kim W., Kim S.M., Bland J.S., Grayson N., Fang M. (2018). Intestinal bitter taste receptor activation alters hormone secretion and imparts metabolic benefits. Mol. Metab..

[67] Kidd M., Modlin I.M., Gustafsson B.I., Drozdov I., Hauso O., Pfragner R. (2008). Luminal regulation of normal and neoplastic human EC cell serotonin release is mediated by bile salts, amines, tastants, and olfactants. Am. J. Physiol. Gastrointest. Liver Physiol..

[68] Wu S.V., Rozengurt N., Yang M., Young S.H., Sinnett-Smith J., Rozengurt E. (2002). Expression of bitter taste receptors of the T2R family in the gastrointestinal tract and enteroendocrine STC-1 cells. Proc. Natl. Acad. Sci. USA.

[69] Kiyofuji A., Yui K., Takahashi K., Osada K. (2014). Effects of xanthohumol-rich hop extract on the differentiation of preadipocytes. J. Oleo Sci..

[70] Miranda C.L., Elias V.D., Hay J.J., Choi J., Reed R.L., Stevens J.F. (2016). Xanthohumol improves dysfunctional glucose and lipid metabolism in diet-induced obese C57BL/6J mice. Arch. Biochem. Biophys..

[71] Zhang Y., Bobe G., Revel J.S., Rodrigues R.R., Sharpton T.J., Fantacone M.L., Raslan K., Miranda C.L., Lowry M.B., Blakemore P.R. (2020). Improvements in metabolic syndrome by xanthohumol derivatives are linked to altered gut microbiota and bile acid metabolism. Mol. Nutr. Food Res..

[72] Yamashita M., Fukizawa S., Nonaka Y. (2020). Hop-derived prenylflavonoid isoxanthohumol suppresses insulin resistance by changing the intestinal microbiota and suppressing chronic inflammation in high fat diet-fed mice. Eur. Rev. Med. Pharmacol. Sci..

[73] Pietschmann P., Rauner M., Sipos W., Kerschan-Schindl K. (2009). Osteoporosis: An age-related and gender-specific disease—A mini-review. Gerontology.

[74] Biino G., Casula L., De Terlizzi F., Adamo M., Vaccargiu S., Francavilla M., Loi D., Casti A., Atzori M., Pirastu M. (2011). Epidemiology of osteoporosis in an isolated sardinian population by using quantitative ultrasound. Am. J. Epidemiol..

[75] Price C.T., Koval K.J., Langford J.R. (2013). Silicon: A review of its potential role in the prevention and treatment of postmenopausal osteoporosis. Int. J. Endocrinol..

[76] Fairweather-Tait S.J., Skinner J., Guile G.R., Cassidy A., Spector T.D., MacGregor A.J. (2011). Diet and bone mineral density study in postmenopausal women from the twinsUK registry shows a negative association with a traditional english dietary pattern and a positive association with wine. Am. J. Clin. Nutr..

[77] Marrone J.A., Maddalozzo G.F., Branscum A.J., Hardin K., Cialdella-Kam L., Philbrick K.A., Breggia A.C., Rosen C.J., Turner R.T., Iwaniec U.T. (2012). Moderate alcohol intake lowers biochemical markers of bone turnover in postmenopausal women. Menopause.

[78] Liel Y., Shany S., Smirnoff P., Schwartz B. (1999). Estrogen increases 1,25-dihydroxyvitamin D receptors expression bioresponse in the rat duodenal mucosa. Endocrinology.

[79] Tucker K.L. (2009). Osteoporosis prevention and nutrition. Curr. Osteoporos. Rep..

[80] Sommer I., Erkkilä A.T., Järvinen R., Mursu J., Sirola J., Jurvelin J.S., Kröger H., Tuppurainen M. (2013). Alcohol consumption and bone mineral density in elderly women. Public Health Nutr..

[81] McLernon D.J., Powell J.J., Jugdaohsingh R., Macdonald H.M. (2012). Do lifestyle choices explain the effect of alcohol on bone mineral density in women around menopause?. Am. J. Clin. Nutr..

[82] Yin J., Winzenberg T., Quinn S., Giles G., Jones G. (2011). Beverage-specific alcohol intake and bone loss in older men and women: A longitudinal study. Eur. J. Clin. Nutr..

[83] Mukamal K.J., Robbins J.A., Cauley J.A., Kern L.M., Siscovick D.S. (2007). Alcohol consumption, bone density, and hip fracture among older adults: The cardiovascular health study. Osteoporos. Int..

[84] Pedrera-Zamorano J.D., Lavado-Garcia J.M., Roncero-Martin R., Calderon-Garcia J.F., Rodriguez-Dominguez T., Canal-Macias M.L. (2009). Effect of beer drinking on ultrasound bone mass in women. Nutrition.

[85] Kubo J.T., Stefanick M.L., Robbins J., Wactawski-Wende J., Cullen M.R., Freiberg M., Desai M. (2013). Preference for wine is associated with lower hip fracture incidence in post-menopausal women. BMC Womens Health.

[86] Berg K.M., Kunins H.V., Jackson J.L., Nahvi S., Chaudhry A., Harris K.A., Malik R., Arnsten J.H. (2008). Association between alcohol consumption and both osteoporotic fracture and bone density. Am. J. Med..

[87] Hansen S.A., Folsom A.R., Kushi L.H., Sellers T.A. (2000). Association of fractures with caffeine and alcohol in postmenopausal women: The Iowa Women’s Health Study. Public Health Nutr..

[88] Hardcastle A.C., Aucott L., Reid D.M., MacDonald H.M. (2011). Associations between dietary flavonoid intakes and bone health in a scottish population. J. Bone Miner. Res..

[89] Welch A., MacGregor A., Jennings A., Fairweather-Tait S., Spector T., Cassidy A. (2012). Habitual flavonoid intakes are positively associated with bone mineral density in women. J. Bone Miner. Res..

[90] Effenberger K.E., Johnsen S.A., Monroe D.G., Spelsberg T.C., Westendorf J.J. (2005). Regulation of osteoblastic phenotype and gene expression by hop-derived phytoestrogens. J. Steroid Biochem. Mol. Biol..

[91] Xia T.S., Lin L.Y., Zhang Q.Y., Jiang Y.P., Li C.H., Liu X.Y., Qin L.P., Xin H.L. (2019). Humulus lupulus, L. extract prevents ovariectomy-induced osteoporosis in mice and regulates activities of osteoblasts and osteoclasts. Chin. J. Integr. Med..

[92] Tobe H., Muraki Y., Kitamura K., Komiyama O., Sato Y., Sugioka T., Maruyama H.B., Matsuda E., Nagai M. (1997). Bone resorption inhibitors from hop extract. Biosci. Biotechnol. Biochem..

[93] Lambert M.N.T., Hu L.M., Jeppesen P.B. (2017). A systematic review and meta-analysis of the effects of isoflavone formulations against estrogen-deficient bone resorption in peri- and postmenopausal women. Am. J. Clin. Nutr..

[94] Prouillet C., Mazière J.-C., Mazière C., Wattel A., Brazier M., Kamel S. (2004). Stimulatory effect of naturally occurring flavonols quercetin and kaempferol on alkaline phosphatase activity in MG-63 human osteoblasts through ERK and estrogen receptor pathway. Biochem. Pharmacol..

[95] Jeong H.M., Han E.H., Jin Y.H., Choi Y.H., Lee K.Y., Jeong H.G. (2011). Xanthohumol from the hop plant stimulates osteoblast differentiation by RUNX2 activation. Biochem. Biophys. Res. Commun..

[96] Luo D., Kang L., Ma Y., Chen H., Kuang H., Huang Q., He M., Peng W. (2014). Effects and mechanisms of 8-prenylnaringenin on osteoblast MC3T3-E1 and osteoclast-like cells RAW264.7. Food Sci. Nutr..

[97] Dong M., Jiao G., Liu H., Wu W., Li S., Wang Q., Xu D., Li X., Liu H., Chen Y. (2016). Biological silicon stimulates collagen type 1 and osteocalcin synthesis in human osteoblast-like cells through the BMP-2/Smad/RUNX2 signaling pathway. Biol. Trace Elem. Res..

[98] Boguszewska-Czubara A., Pasternak K. (2011). Silicon in medicine and therapy. J. Elem..

[99] Suh K.S., Rhee S.Y., Kim Y.S., Lee Y.S., Choi E.M. (2013). Xanthohumol modulates the expression of osteoclast-specific genes during osteoclastogenesis in RAW264.7 cells. Food Chem. Toxicol..

[100] Chen Y.-M., Ho S.C., Lam S.S.H., Ho S.S.S., Woo J.L.F. (2003). Soy isoflavones have a favorable effect on bone loss in chinese postmenopausal women with lower bone mass: A double-blind, randomized, controlled trial. J. Clin. Endocrinol. Metab..

[101] Giersch G.E.W., Charkoudian N., Stearns R.L., Casa D.J. (2020). Fluid balance and hydration considerations for women: Review and ruture directions. Sport. Med..

[102] Stachenfeld N.S., DiPietro L., Palter S.F., Nadel E.R. (1998). Estrogen influences osmotic secretion of AVP and body water balance in postmenopausal women. Am. J. Physiol. Regul. Integr. Comp. Physiol..

[103] Stachenfeld N.S., Splenser A.E., Calzone W.L., Taylor M.P., Keefe D.L. (2001). Selected contribution: Sex differences in osmotic regulation of AVP and renal sodium handling. J. Appl. Physiol..

[104] González-SanJosé M.L., Rodríguez P.M., Valls-Bellés V. (2017). Beer and its role in human health. Fermented Foods in Health and Disease Prevention.

[105] Jiménez-Pavón D., Cervantes-Borunda M.S., Díaz L.E., Marcos A., Castillo M.J. (2015). Effects of a moderate intake of beer on markers of hydration after exercise in the heat: A crossover study. J. Int. Soc. Sports Nutr..

[106] Orrù S., Imperlini E., Nigro E., Alfieri A., Cevenini A., Polito R., Daniele A., Buono P., Mancini A. (2018). Role of functional beverages on sport performance and recovery. Nutrients.

[107] Hobson R.M., Maughan R.J. (2010). Hydration status and the diuretic action of a small dose of alcohol. Alcohol Alcohol..

[108] Polhuis K.C.M.M., Wijnen A.H.C., Sierksma A., Calame W., Tieland M. (2017). The diuretic action of weak and strong alcoholic beverages in elderly men: A randomized diet-controlled crossover trial. Nutrients.

[109] Shirreffs S.M., Maughan R.J. (2006). The effect of alcohol on athletic performance. Curr. Sports Med. Rep..

[110] Barnes M.J., Mündel T., Stannard S.R. (2011). A low dose of alcohol does not impact skeletal muscle performance after exercise-induced muscle damage. Eur. J. Appl. Physiol..

[111] Shirreffs S.M., Maughan R.J. (1997). Restoration of fluid balance after exercise-induced dehydration: Effects of alcohol consumption. J. Appl. Physiol..

[112] Flores-Salamanca R., Aragón-Vargas L.F. (2014). Postexercise rehydration with beer impairs fluid retention, reaction time, and balance. Appl. Physiol. Nutr. Metab..

[113] Castro-Sepulveda M., Johannsen N., Astudillo S., Jorquera C., Álvarez C., Zbinden-Foncea H., Ramírez-Campillo R. (2016). Effects of beer, non-alcoholic beer and water consumption before exercise on fluid and electrolyte homeostasis in athletes. Nutrients.

[114] Wijnen A.H.C., Steennis J., Catoire M., Wardenaar F.C., Mensink M. (2016). Post-exercise rehydration: Effect of consumption of beer with varying alcohol content on fluid balance after mild dehydration. Front. Nutr..

[115] Desbrow B., Murray D., Leveritt M. (2013). Beer as a sports drink? Manipulating beer’s ingredients to replace lost fluid. Int. J. Sport Nutr. Exerc. Metab..

[116] Desbrow B., Cecchin D., Jones A., Grant G., Irwin C., Leveritt M. (2015). Manipulations to the alcohol and sodium content of beer for postexercise rehydration. Int. J. Sport Nutr. Exerc. Metab..

